# Manipulation under anaesthesia versus arthroscopic capsular release in stiff shoulder: A prospective multicentre study

**DOI:** 10.17159/2078-516X/2026/v38i1a25790

**Published:** 2026-08-15

**Authors:** AS Abutaleb, AI Eltarawy, MT Elshewy, H Amin

**Affiliations:** 1 Department of Orthopaedic Surgery, Faculty of Medicine, Cairo University, Cairo, Egypt; 2 El-Sahel Teaching Hospital, Cairo, Egypt

**Keywords:** Constant score, Frozen Shoulder, Adhesive capsulitis

## Abstract

**Background:**

Manipulation under anaesthesia (MUA) and arthroscopic capsular release (ACR) are common modalities of management in shoulder stiffness, yet no robust clinical evidence demonstrates the superiority of one over the other.

**Objectives:**

Comparing functional results, complications, and operative time between MUA and ACR.

**Methods:**

In this prospective multicentre cohort study, 62 patients with primary shoulder stiffness of at least 6 months’ duration, forward elevation less than 100°, and external rotation limited by more than 50% compared with the contralateral side, despite conservative management, were included. Exclusion criteria were glenohumeral osteoarthritis, periarticular fractures, mechanical block, and skeletal immaturity. Patients treated in centre 1 underwent MUA (group A: n=31) and those in centre 2 underwent ACR (group B: n=31), with standardised anaesthesia, rehabilitation, and follow-up at 4 and 6 months.

**Results:**

Baseline demographics were comparable between groups. Both MUA and ACR produced significant improvements in Constant score, VAS, and ROM across all planes at 4 and 6 months, with no significant between-group differences. No major intra-operative or post-operative complications were observed in either cohort during the 6-month follow-up period. The operative time was significantly lower in the MUA group.

**Conclusion:**

MUA and ACR are both effective in improving pain and shoulder function in refractory shoulder stiffness, but MUA offers a shorter operative time. In the management of shoulder stiffness, MUA has comparable functional outcomes with ACR but with shorter operative duration.

Adhesive capsulitis is a common problem in orthopaedic practice; its incidence is estimated at 24 patients per 10000 population per year^[1]^ with a prevalence of 2–5% of the population.^[2]^ Affected patients usually complain of a sudden onset of pain with significant restriction of both active and passive shoulder range of motion (ROM). Severe pain can be expected, especially at night.^[2,3]^ To decrease time to recovery and improve outcomes, a variety of regimens have been used for the treatment of primary adhesive capsulitis, which include non-steroidal anti-inflammatory drugs (NSAIDs), local intra-articular steroid injection, physiotherapy, hydro-dilation, manipulation under anaesthesia (MUA) and arthroscopic capsular release (ACR).^[4]^

MUA has been a long-standing treatment for shoulder stiffness. Possible complications of this procedure include proximal humerus fracture, brachial plexus injury, hemarthrosis, haemorrhage, and tearing of the capsule, glenohumeral ligament, glenoid labrum, and the rotator cuff.^[5,6]^ While ACR, as a modality of treatment for shoulder stiffness, has the advantage of being a more controlled release for capsule restraints, it carries the risk of injury to the axillary nerve, postoperative shoulder instability, dislocations, and chondrolysis.^[5,7]^

Numerous studies have compared ACR and MUA as treatment modalities for shoulder stiffness, yet no robust clinical evidence demonstrates the superiority of either. Reported outcomes have been heterogeneous, revealing either no significant differences in range of motion (ROM), pain scores, or complication rates^[8,9]^; an elevated short-term risk of complications associated with ACR^[9]^ or accelerated improvement in forward flexion with ACR.^[10]^ Conversely, certain investigations have favoured MUA for earlier gains in forward flexion, external/internal rotation, and Visual Analogue Scale (VAS) scores.^[4]^

In the current study, we hypothesise that in patients with primary adhesive capsulitis, MUA can achieve shoulder function comparable to ACR but with a shorter operative time. The primary outcome was the change in Constant–Murley score^[11]^ at 6 months, with operative time, pain (VAS),^[12]^ ROM parameters and incidence of complications treated as secondary outcomes, and 6 months specified as the primary assessment time point.

## Methods

### Study design and ethical clearance

This prospective study was approved by the Ethical Committee of Cairo University Hospitals, Cairo, Egypt (approval code: MD-321-2023). Written informed consent was obtained from all participants.

### Participants and sampling

Between July 2023 and June 2025, patients with shoulder stiffness were consecutively enrolled if they exhibited restricted active and passive shoulder range of motion (ROM), defined as forward elevation <100° and external rotation >50% reduced compared with the contralateral side, with no improvement after ≥6 months of conservative management with physiotherapy and NSAIDs. Exclusion criteria included glenohumeral osteoarthritis, periarticular fractures, mechanical block as the cause of stiffness, and skeletal immaturity.

Sixty-six patients were initially enrolled in the study. Four patients did not complete the required follow-up protocol and were excluded (three in group A and one in group B); thus, the final analysis included 62 patients. Patients were divided into two groups based on surgical intervention: Group A underwent MUA, and Group B underwent ACR. Surgical procedures were performed by the 1^st^, 2^nd^, and 3^rd^ authors across two centres, with each surgeon affiliated with all participating centres. Patient allocation to treatment was centre-based and non-randomised: patients admitted to centre 1 underwent MUA (group A), while those admitted to centre 2 underwent ACR (group B). Although both groups followed standardised pre- and postoperative protocols, this centre-based allocation introduces potential centre-level confounding that should be considered when interpreting the findings.

### Surgical techniques

All procedures were conducted under general anaesthesia combined with interscalene regional block.

*Group A (MUA):* With the patient supine, the operator grasped the proximal humerus near the axilla with one hand while stabilising the scapula with the other to create a short lever arm and isolate the glenohumeral joint. Manipulation proceeded sequentially: forward elevation, abduction, external rotation, internal rotation, and adduction. During gradual manipulation, an audible click was heard. The manipulation force was gradually increased until full passive ROM was achieved as compared to the contralateral side. Neither fluoroscopy nor arthroscopy was used during the procedure.

*Group B (ACR):* With the patient in the beach-chair position, a 360° capsular release was performed via standard arthroscopic portals. Sequential release targeted the rotator interval, coracohumeral ligament, middle glenohumeral ligament, anterior inferior glenohumeral ligament, and posterior inferior glenohumeral ligament. To protect the axillary nerve, the following was done: the arm was placed in slight flexion, and the ACR was performed sequentially, layer by layer, while restricting the release below the glenoid. A radiofrequency ablation device was used to perform controlled tissue dissection with continuous saline irrigation to prevent thermal injury.

### Postoperative management and follow-up

Postoperatively, in both groups, an arm sling and ice packs were applied for 2 days. All patients received identical NSAIDs for 2 weeks. Physiotherapy was started immediately after the surgery. It consisted of 3 weekly sessions along with daily home exercises. The first 6 weeks involved passive ROM, followed by 6 weeks of active ROM. Finally, shoulder-strengthening exercises were performed for 12 weeks.

Follow-up visits were scheduled at 2 days, 1 week, 2 weeks, and 2, 4, and 6 months postoperatively to monitor physiotherapy progression and record outcome variables. Patients' adherence to the follow-up protocol was measured via patient-completed exercise logs (target >80% compliance). An attendance/performance sheet for supervised sessions and prescribed exercises was reviewed by the investigators at the end of follow-up to confirm patients’ adherence.

### Outcome measures

The primary outcome of the study was the Constant Shoulder Score at 6 months. Further secondary outcome parameters included the VAS, the surgical procedure duration, ROM, encompassing forward elevation, shoulder abduction, external rotation in both adducted and abducted shoulders, and internal rotation in both adducted and abducted shoulders. Secondary outcomes also involved the occurrence of complications.

For the pain component, we used a 10 cm visual analogue scale (VAS). ROM was measured with a standard universal goniometer by the centre physiotherapist, with the patient seated, the scapula stabilised as far as possible, and the limb positioned according to commonly used goniometric guidelines. The same physiotherapist at each centre performed repeated assessments throughout the study, and all assessors received pre-study instruction on the measurement protocol, reflecting the high intra and inter-rater reliability reported for shoulder ROM goniometry when standardised procedures are used. Internal rotation in adduction was recorded categorically using the highest spinal level reached by the patient’s thumb behind the back, and this was approximated to the nearest level aligning with the constant score internal rotation category levels: Lateral thigh, Behind the buttock, Sacroiliac joint, Waist, 12th Thoracic vertebra.

Complications were prospectively monitored and classified as either intra operative (including humeral fracture, glenohumeral dislocation, iatrogenic rotator cuff or labral tear requiring treatment, neurovascular injury, and anaesthetic related adverse events requiring intervention) or post operative up to 6 months (including wound problems or deep infection, thromboembolic events, post operative instability or dislocation, cuff tear, chondrolysis, unplanned re operation or readmission, or any other deviation from the expected postoperative course judged by the treating surgeon to be at least possibly related to the index procedure and requiring additional treatment, unplanned imaging, or modification of rehabilitation).

Constant score and shoulder ROM were first recorded in the outpatient clinic by the centre physiotherapist using standardised assessment techniques. The first author then independently checked these data, and any discrepancies were resolved by consensus with the second author. Formal blinding of outcome assessors was not feasible, as both the treating teams and physiotherapists were necessarily aware of the procedure performed. Data were recorded at 4 and 6 months.

### Statistical analysis

The sample size was calculated based on the effect size reported in previous studies.^[4]^ Statistical analysis was done by SPSS v26 (IBM Inc., Chicago, IL, USA). The Shapiro-Wilk test and histograms were used to assess the normality of the data distribution. Quantitative non-parametric data were presented as median and range, and Hodges–Lehmann median differences were reported (with 95% CIs) alongside the non-parametric tests. Qualitative variables were presented as frequencies and percentages (%) and analysed using the Chi-square test or Fisher's exact test, as appropriate. No imputation was performed for missing outcome data; the analyses used only complete cases. A two-tailed P value < 0.05 was considered statistically significant.

## Results

Patients’ demographic data showed no significant difference between the two groups. Comorbidities were similar between groups: diabetes mellitus was present in 18 patients (58%) in group A and 20 patients (65%) in group B (p = 0.602), while hypertension occurred in 17 patients (55%) in group A and 15 patients (49%) in group B (p = 0.611) (Table 1).

Regarding intra-operative variables, the median (range) surgery time in group A, 18 (15–40) min, was significantly lower than in group B, 67 (45–120) min, p <0.001 (Table 2).

Post-operatively, both groups showed significant improvements in Constant Score, VAS, and ROM at all shoulder planes of movement at 4 and 6 months, with no significant differences between the two groups (Table 2).

Changes from baseline and median between-group differences were compared with 95% confidence intervals (Table 2). The mean (± SD) follow-up adherence was 90 ± 5% in Group A and 90 ± 6% in Group B.

No intra-operative complications (Iatrogenic tear, fracture, dislocation or anaesthetic-related events) were recorded in either the MUA or ACR cohorts. No post-operative wound or deep infections, thromboembolic events, post-operative instability/dislocation, neurovascular injury, unplanned re-operations, or unplanned readmissions related to the index procedure were observed in either group during the 6-month follow-up (all categories 0/31 in each group).

## Discussion

The main outcome of the study is that, in patients with shoulder stiffness, MUA achieved a shoulder Constant score improvement comparable to ACR at 4 and 6 months, with significantly lower operative time.

Adhesive capsulitis is a common, disabling disorder that is initially treated with conservative measures. MUA and ACR were effectively used in the management of refractory cases, with ongoing debate over which procedure is more reliable/effective in their treatment. We conducted a prospective cohort study to compare the two procedures with respect to functional recovery at different postoperative follow-up time points, with particular focus on operative duration.

The current study included 62/66 patients. Only patients with complete data (62 patients) were included in the final statistical tests (complete case analysis).

The study was designed as a pragmatic, prospective multicentre cohort rather than a randomised trial, primarily to reflect real-world practice patterns and to accommodate differing institutional preferences for MUA or ACR. rendering the current findings interpreted as observational comparative effectiveness data rather than definitive causal estimates.

According to our findings, Constant score, shoulder ROM, and VAS improved significantly at 4 and 6 months postoperatively with no significant difference between the two groups. However, the MUA showed significantly lower surgical time. No correction for multiple testing was performed, acknowledging the consequent risk of inflated Type I error for secondary endpoints, and emphasising that p-values for these outcomes should be interpreted cautiously and in conjunction with effect sizes, confidence intervals, and clinical plausibility rather than as stand-alone proof of effect.

Sundararajan et al.^[13]^ conducted a RCT comparing both techniques and showed similar results to ours at 24 weeks of follow-up. But they didn’t record earlier functional outcome and did not evaluate the surgical time.

Zhao et al.^[9]^ showed in a systematic review and meta-analysis that ROM improvements did not differ between ACR and MUA groups across follow-up time points, with significantly higher risk of complications in the ACR group. They did not calculate the surgery time.

In a meta-analysis, Xiao et al.^[10]^ found no significant differences between ACR and MUA in terms of post-operative external rotation and forward elevation at 12-month follow-up, with comparable complication risk. However, they found statistically significant differences between ACR and MUA for forward elevation at 3 and 6 months. The surgery time was not recorded. Kim et al.^[4]^ found that the MUA group showed significantly greater shoulder forward elevation at 3 months, but the mean external rotation and internal rotation were significantly greater at 3, 6, and 12 months after the procedure. They used the American Shoulder and Elbow Surgeons (ASES) score for functional evaluation, with no referral to surgery time.

Uluyardimci et al. and Lee et al.^[8,14]^ reported similar results regarding functional outcome using the ASES score. But their studies were retrospective and Uluyardimci et al had a smaller sample size (32 patients).

The findings of this study should be interpreted with caution due to several methodological limitations. Treatment allocation was determined by centre rather than by randomisation, and outcome assessors and therapists were not blinded. Consequently, subtle between-centre differences in rehabilitation delivery remain potential sources of performance bias. The follow-up duration was limited to six months, which may result in missed detection of long-term or very late complications or additional procedures. The study was also underpowered to detect rare events and minor complications. Several important baseline characteristics—including glycaemic control, symptom duration, and prior conservative therapy—were incompletely recorded, introducing the possibility of residual confounding. Although adherence to the planned protocol appeared generally high and comparable between groups at the aggregate level, the available adherence data may not fully capture qualitative differences in physiotherapy delivery. Furthermore, while the proportion of patients lost to follow-up was small (4/66) and baseline characteristics showed no major differences, this attrition could still introduce some risk of bias if those lost differed systematically from those retained.

## Conclusion

Both MUA and ACR were associated with improved outcomes over 6 months; no between-group difference in measured clinical outcomes was observed in this cohort; MUA had a shorter operative duration.

## Figures and Tables

**Table 1:** Baseline characteristics of the study cohort (n=62)

		Group A (MUA) (n=31)	Group B (ACR) (n=31)	p-value
Age	Median (Range)	52 (36 – 70)	48 (35 – 67)	0.607
Sex	Male	14 (45%)	9 (29%)	0.189
	Female	17 (55%)	22 (71%)	
Co-morbidities	Diabetes mellitus	18 (58%)	20 (65%)	0.602
	Hypertension	17 (55%)	15 (49%)	0.611
Affected side	Right	17 (55%)	17 (55%)	1.0
	Left	14 (45%)	14 (45%)	

**Data** expressed as median (range) or count and percentage of group total. Participant could have both co-morbidities. P-value reported for sex (male vs female) and affected side (right vs left) not groups. MUA, Manipulation under anaesthesia; ACR, Arthroscopic Capsular Release.

**Table 2:** Clinical and functional outcomes of the two treatment groups (n=62)

	Group A (MUA) (n=31)	Group B (ACR) (n=31)	p-value	Between-group differences		

				Median difference	95% CI	
	
	Median (range)	Median (range)			Upper	Lower

**Duration of surgery (min)**	18 (15–40)	67 (45–120)	<0.001	−52	−52	−49

**Constant score**						
Pre-op	26 (22–40)	26 (18–35)	0.426	1	−2	5
4 months post-op	66 (60–88)	67 (60–84)	0.457	1	−2	4
6 months post-op	82 (66–91)	77 (72–93)	0.347	3	−2	6
Change from baseline	53 (33–96)	52 (48–68)	0.983	0	−3	3

**Visual Analogue Scale (VAS) score**						
Pre-op	7 (4–10)	8 (5–10)	0.470	0	−1	1
4 months post-op	3 (1–6)	4 (2–6)	0.348	0	−1	0
6 months post-op	3 (1–4)	2 (1–5)	0.894	0	−1	0
Change from baseline	4 (2–9)	5 (4–8)	0.226	0	−1	0

**Forward elevation**						
Pre-op	90 (80–115)	90 (70–100)	0.566	0	−5	0
4 months post-op	140 (120–180)	140 (120–180)	0.603	0	−10	10
6 months post-op	170 (135–180)	170 (135–180)	0.287	0	0	10
Change from baseline	80 (40–100)	70 (45–100)	0.386	0	0	10

**Abduction**						
Pre-op	70 (60–90)	70 (65–90)	0.346	0	−5	0
4 months post-op	130 (90–170)	135 (90–170)	0.129	−5	−15	0
6 months post-op	155 (120–180)	150 (90–170)	0.344	−5	−15	5
Change from baseline	75 (50–110)	80 (10–105)	0.320	−5	−15	5

**External rotation (in abduction)**						
Pre-op	35 (20–70)	25 (10–60)	0.509	5	−5	10
4 months post-op	65 (30–90)	65 (30–90)	0.994	0	−5	5
6 months post-op	85 (70–90)	80 (70–90)	0.445	0	0	0
Change from baseline	50 (20–65)	55 (30–80)	0.204	−5	−5	0

**External rotation (in adduction)**						
Pre-op	20 (10–30)	20 (0–30)	0.382	0	0	0
4 months post-op	60 (20–90)	55 (30–80)	0.365	5	−5	10
6 months post-op	75 (50–90)	70 (50–90)	0.808	0	−5	5
Change from baseline	50 (30–70)	50 (20–75)	0.621	0	−5	5

**Internal rotation (in abduction)**						
Pre-op	20 (10–50)	20 (10–50)	0.815	0	0	5
4 months post-op	60 (20–80)	50 (30–75)	0.689	0	−5	10
6 months post-op	70 (50–90)	65 (50–80)	0.173	5	0	10
Change from baseline	40 (20–70)	45 (20–70)	0.831	0	−5	10

**Internal rotation (in adduction)**						
***Pre-op***						
Lateral thigh	10 (32%)	10 (32%)	0.891	NA	NA	NA
Behind buttock	11 (36%)	13 (42%)				
Sacroiliac joint	5 (16%)	5 (16%)				
Waist	5 (16%)	3 (10%)				
12th thoracic vertebra	0	0				

***4 months post-op***						
Lateral thigh	0	0	0.896	NA	NA	NA
Behind buttock	0	0				
Sacroiliac joint	12 (39%)	13 (42%)				
Waist	11 (36%)	12 (39%)				
12th thoracic vertebra	8 (26%)	6 (19%)				

***6 months post-op***						
Lateral thigh	0	0	0.39	NA	NA	NA
Behind buttock	0	0				
Sacroiliac joint	0	0				
Waist	8 (26%)	10 (32%)				
12th thoracic vertebra	23 (74%)	21 (68%)				

95% confidence interval for the median difference using the Hodges-Lehmann estimation. MUA, Manipulation under anaesthesia; ACR, Arthroscopic Capsular Release; NA, not applicable as variables were categorical.
